# Clinical ethics: medical tourism in children

**DOI:** 10.1136/archdischild-2021-322778

**Published:** 2021-09-22

**Authors:** Giles Birchley, Mike Linney, Stephen W Turner, Dominic Wilkinson

**Affiliations:** 1 Centre for Ethics in Medicine, Bristol Medical School, Bristol, UK; 2 Women And Childrens, Western Sussex Hospitals NHS Foundation Trust, Worthing, West Sussex, UK; 3 Department of Child Health, University of Aberdeen, Aberdeen, UK; 4 Oxford Uehiro Centre for Practical Ethics, University of Oxford, Oxford, UK

**Keywords:** ethics, global health

Paediatricians sometimes learn that parents plan to take a child overseas for medical treatment (box 1). How should they respond?

Box 1 **Cases of medical tourism in children**

- A child with severe eczema whose parents are first-generation immigrants. Parents plan to return to their home country for a second opinion.
- A child with relapsed malignancy with a short time to live. Parents are planning to take abroad for an experimental vitamin therapy.
- A child with autism. Parents plan to take overseas for intrathecal stem cell therapy.
- A young person with complex epilepsy. Parents planning to take overseas for cannabinoid-based medications.
- A child in a minimally conscious state, ventilated in intensive care. Parents wish to take to another country for tracheostomy and ongoing intensive care.
- A girl with no medical conditions. Parents plan to take overseas for female circumcision.

Child medical tourism is ‘the bi-directional movement of children … to and from a country to seek advice, diagnosis and treatments’.^1^ In the UK, it is estimated that (pre-COVID-19) 63 000 adult patients sought treatment abroad yearly. The number of children (patients <18 years) involved in medical tourism is unknown. Decisions to seek treatment abroad are made privately by parents and usually uncontested by National Health Service (NHS) staff, despite some high-profile court cases.

Child medical tourism happens because parents prefer another healthcare system (case A, box 1) or want access to therapies unavailable in the UK. Therapies may be unavailable because they are experimental (B, C), unlicensed for the indication or parents are unable to find a willing prescriber (D), there is disagreement about the child’s best interests (E), or they are illegal (F). Treatments range from those involving entirely unverified technologies to others where some evidence exists but below best evidence level. Where a ‘medically justifiable’ therapy is unavailable in the UK, some funding may be found through the NHS (via the ‘S2’ funding scheme). In many cases parents fund the treatment privately.

## Areas of concern

Health tourism has been lauded as a means by which low-income and middle-income countries can grow and develop strong service economies. Nevertheless there are costs for local populations who are usually unable to access hospitals and clinicians serving health tourists. While inbound and outbound medical tourism for adults has been estimated to be cost-neutral to the NHS,^2^ paediatric provision has not been modelled. Complex long-term treatments that are initiated abroad could have significant resource implications to the NHS (for example tracheostomy and home ventilation).

Cultures vary in their attitudes towards children and the appropriate limits of medical treatment. With variable accreditation, the quality of accessed healthcare treatments also varies. Adult patients have reported high satisfaction following treatment overseas, although lack of long-term follow-up may mask longer-term problems. Obtaining redress in cases of treatment failure or negligence is tricky, and receiving corrective treatment on the NHS after the child returns home is not guaranteed. Treatment abroad often lacks continuity of care, with variable (or absent) sharing of medical information between countries if there are problems.

Because medical treatment is expensive, crowdfunding websites have become a popular method of raising funds. The emphasis on public sympathy means that crowdfunding does not distribute resources equitably, and minority groups may raise less funds through crowdfunding than their non-minority peers.^3^ Crowdfunding publicises the private health information of a child with potentially lifelong impacts. Where parents are seeking an ‘innovative’ or unusual treatment, there is little consensus about the standards of acceptable treatment in case law. Notionally, an acceptable treatment must be adequate on the grounds of evidence, expertise and infrastructure,^4^ yet the courts have sometimes adopted lower standards.

Despite these concerns, and notwithstanding the risks, seeking treatment for a child abroad is not in itself unreasonable. Paediatricians should engage with, educate and support parents to make informed decisions in the best interests of the child.

## Recommendations

Decisions about medical treatment for children are based on their best interests, arrived at through a process of shared decision-making with the child’s parents/caregivers. Paediatricians and primary care professionals should encourage parents to discuss their hopes and plans for their child. Such discussions are only likely if parents perceive the health professionals as open-minded, supportive and willing to engage in dialogue. Clinicians should empathically explore parents’ understanding of a child’s illness and prognosis, their reasons for seeking treatment abroad, and their priorities and concerns.

Doctors in the UK should advise parents and support them where appropriate in identifying reputable institutions to perform treatment. A reputable institution should have i) Clinicians experienced in giving the proposed therapy; ii) A clear, evidence-based, treatment plan and a proven ability to deliver, and; iii) A plan for aftercare and long-term follow-up. A reputable overseas provider will also have no problem with involving the parents in planning treatment and forming relationships with UK clinicians to deliver long-term follow-up. Where families seeking overseas care are making a reasonable choice, clinicians should support best medical care by referring directly to the overseas institution. Where there are poor standards of information from institutions whose business is medical tourism, these institutions should be treated with suspicion. Parents should be encouraged to find different providers if there are concerns about an institution’s approach.

Without jeopardising engagement with parents, doctors should ensure that parents are aware of their limited legal redress if things go wrong, even from ‘safe’ destinations within the European Economic Area and USA. There is no guarantee that overseas institutions will cooperate with the courts or agree that a case for damages will be heard in the UK. Even if it is, UK courts are obliged to follow the laws of the destination country. There may be weaker protections in negligence cases, with low caps on damages or limits on how long compensation may be claimed. Where damages are awarded, it may be impossible to compel overseas institutions to pay them. Depending on what went wrong, it could be difficult to get problems treated on the NHS.^5^ Furthermore, even where treatment commenced overseas is successful it may not be funded on the child/young person’s return to the UK (although the best interests of the child will be the deciding factor in these cases). Parents should be advised of these difficulties so they can make an informed choice.

Sometimes parents will remain fixed on a treatment that a UK doctor has severe concerns about, perhaps because a doctor at the destination makes unlikely claims, a treatment is ineffective, appears dangerous or imposes burdens on the child. A key ethical consideration is whether the proposed course of action exposes the child to risks of harm that are disproportionate to any benefits. If the travel to the proposed destination, treatment and aftercare is of questionable benefit, but low risk, it may be reasonable for parents to pursue it.

Clinicians have a legal duty of care towards the child. As well as satisfying themselves of the reputability of a foreign institution, it may be helpful to seek a second opinion, in order to provide parents with an alternative point of view and clarify harms and benefits of the proposed treatment. Where the clinician remains concerned about the child’s well-being, and engagement and second opinions fail, it may be appropriate to involve the courts. Occasionally parents will want a treatment that is criminal (eg, results in significant and foreseeable harms). Parents should be aware that circumventing the law will result in prosecution when it comes to light, and clinicians must take immediate action to protect the child.

## Conclusion

Seeking medical treatment abroad is increasingly common and may become more so with the emergence of therapies for rare diseases that are expensive and limited in availability. Medical tourism raises a range of ethical concerns, but it is not necessarily unreasonable to seek treatment for a child abroad. Parents should be supported to make wise and informed choices and to ensure that the interests of the child are at the centre of any decision.
